# Genetic Dissection of the Mixing Properties of Wheat Flour (*Triticum aestivum* L.) Using Unconditional and Conditional QTL Mapping

**DOI:** 10.7150/jgen.67253

**Published:** 2022-01-01

**Authors:** Haixia Yu, Yuling An, Aiping Wang, Xin Guan, Jichun Tian, Tangyuan Ning, Kexin Fan, Hao Li, Qianqian Liu, Dongxue Wang, Jiansheng Chen

**Affiliations:** 1 State Key Laboratory of Crop Biology/Key Laboratory of Crop Water Physiology and Drought-tolerance Germplasm Improvement, Ministry of Agriculture/Group of Wheat Quality Breeding, Shandong Agricultural University, Tai'an 271018, P.R. China.; 2 Dezhou Agricultural Protection and Technological Extension Center, Dezhou, 253000, P.R. China.

**Keywords:** flour mixing properties, unconditional QTL mapping, conditional QTL mapping, HMW-GS

## Abstract

Wheat (*Triticum aestivum* L.) flour mixing properties are essential quality parameters in the dough development process. Limited research on superior alleles for mixing properties has restricted their molecular improvement, and other factors related to the complex traits have been ignored. A molecular map of 9576 polymorphic markers in the RIL population (F_8:9_) (Shannong01-35/Gaocheng9411) was constructed to evaluate mixing property effects in three environments. The parents were selected with markedly distinct high-molecular-weight glutenin subunits (HMW-GS). This study not only evaluated mixing properties using conventional unconditional QTL mapping but also evaluated the relationships between protein-related traits using conditional QTL mapping. The analyses identified most additive QTLs for major mixing properties on chromosomes 1A, 1B, and 1D. Two major loci (*1A.1-15* and *1D-1*) associated with mixing properties have confirmed the important contributions of Glu-A1 and Glu-D1 to wheat quality at the QTL level, which were mainly affected by the gluten index. Another important locus, *1B.1-24* (associated with midline peak value and midline peak width, with high phenotypic variations explained), might represent a new variation distinct from* Glu-B1.* The favored alleles came from Gaocheng9411. Several mixing properties shared the same QTLs (*1B.1-6* and *1A.1-15*), indicating tight linkage or pleiotropism. Genotype-by-environment (G×E) interactions were also investigated in the present study. The QTL results in our study may improve our understanding of the genetic interrelationships between mixing properties and protein-related traits.

## Introduction

Wheat flour mixing properties have been widely applied to test the rheological characteristics of food end products, especially in the baking industry 1, 2. They are typically quantitative traits controlled by multiple genes and susceptible to environmental factors. QTLs (quantitative trait loci) for mixing properties were identified on chromosomes 1B, 1D, 3B, 4D, and 5D using a doubled haploid (DH) population 3. Nelson et al. (2006) found QTLs for mixograph properties with strong effects on chromosomes 1AS, 1BS and 6DS 4. QTLs for nine mixing properties were identified on chromosomes 1B, 2A, 2B, 3A, 5B, 5D, 6B and 6D 5. QTLs for mixing time and mixing 8 min width were identified on chromosomes 1B and 1D from 240 RIL lines (F_5:6_) of PH82-2/Neixiang 188 6. Additionally, QTLs for midline peak time, mixing peak value and midline peak width were identified on chromosomes 1A, 1B, 1D, 2A, 6D and 7D 7.

To date, little information on QTL mapping for mixing properties is available, and larger differences among previous reports were mostly caused by genetic populations or maps. Conventional QTL studies do not actually evaluate closely related traits and the genetic contribution to a single trait. Protein content and quality are strongly impacted by mixing properties. Zhu and Keightley (1995) proposed the conditional genetic analysis method 8. It could study the contributions of single trait to complex traits and the genetic relationships among more than two traits. This method has been widely used to investigate the genetic basis of complex traits in many crops 9-12. In wheat, conditional QTL analyses have also been conducted on plant height 13, kernel weight 14, and protein content 15. At present, few conditional QTL analyses have evaluated the genetic relationships between mixing properties and protein-related traits.

In the present study, both unconditional and conditional QTL mapping were conducted to investigate the underlying genetic basis of mixing properties and to dissect the genetic relationships between these protein-related traits at the QTL level. The results could contribute to marker-assisted breeding for wheat quality, especially for mixing properties.

## Materials and methods

### Plant materials

The RIL population (F_8:9_) with 173 lines was generated by the cross of Shannong01-35 (SN01-35) and Gaocheng9411 (GC9411) 16. The parents were selected with marked distinct high-molecular-weight glutenin subunits (HMW-GS).

### Field trials

The RIL population was harvested at Tai'an (116°36E, 36°57N) in 2009 (E1), 2010 (E2) and Suzhou (116°58E, 33°38N) in 2010 (E3). The materials were planted in a randomized complete block design with two replicates in each environment. Every pot had three rows, and each row was two meters long with a row-to-row distance of 21 centimeters. Crop management was performed according to local practices. The average value of the mixing properties in the above environments was also calculated (AE).

### Measurement of mixing properties and protein-related traits

Harvested seeds were grinded into powder by a Buhler experimental mill (Buhler, Buhler-Miag Co., Germany). The flours were then sealed and kept at 8-10℃ for analysis. Mixing properties were determined by Mixograph (National Mfg. Co., Lincoln, NE) using a 10 g sample of flour system according to AACC-54-40. The mixograph curves (two envelopes and one midline) were computed with Mixsmart software (National Mfg. Co.). Parameters were recorded as follows: MPV (midline peak value), MPW (midline peak width), MPT (midline peak time), EW (end width), MTxV (midline time-8 min value), IT (integral of curve tail), CTV (curve tail value), CTW (curve tail width), SL (left slope of peak) and SR.

Traits PC (protein content), SV (sedimentation volume), WG (wet gluten), DG (dry gluten), and GI (gluten index) were measured. The measurement methods and phenotypic values among the above traits could be viewed in a previous study 16.

### Genotyping and linkage map construction

A total of 9576 polymorphic molecular markers, including 9072 SNPs 17, 18, 442 DArT markers (http://www.triticarte.com.au), 59 SSR markers, 2 biochemical markers and 1 CAPS marker, were used to genotype the RIL population. Genetic linkage groups were constructed using Mapmaker/Exp 3.0. The threshold for marker loci assignment was an LOD score of ≥3.0. The linkage map was ultimately generated using MapChart 2.1 software 19. The genetic map spanned approximately 4048.5 cM, and the average interval of markers was 8.13 cM 16.

### Statistical analysis and QTL mapping

Phenotypic values were analyzed using SPSS 17.0 software. QTL analyses were conducted by QTL Network 2.0 using the mixed-linear model approach. QTLs were designated as follows: *Q*, followed by the abbreviated trait name, then the wheat chromosome on which the QTL was identified; the last number indicated the marker interval on the chromosome. For example, *QMPV1B.1-6* indicated a QTL for MPV identified on the sixth interval of chromosome 1B.1.

## Results

### Phenotypic analysis of mixing properties

The phenotypic values were listed in Table 1. The two parents had marked differences in MPT, CTV, MTxV, CTW and EW. GC9411 exhibited higher parameter values than SN01-35 in all environments. Strong transgressive segregation was detected. This phenomenon may be affected by differences in HMW-GS subunits between parents. Differences in the CV (coefficients of variation) were found for all the traits in all three environments. Among these, CTW possessed the highest CV of 80.32% and a mean of 41.86%, followed by EW, SL and SR, with CV ranges of 37.83-49.27%, 39.46-58.24% and 26.82-36.97%, respectively. The CVs of MPV and MTxV were less than 10%, indicating that these three parameters had little potential for improvement. According to the analysis, the traits followed a normal distribution and were suitable for QTL analysis.

### QTLs for mixing properties

#### Additive QTLs and genotype-by-environment interactions (G×E) for mixing properties MPV, CTV, and MTxV

**Figure 1:**
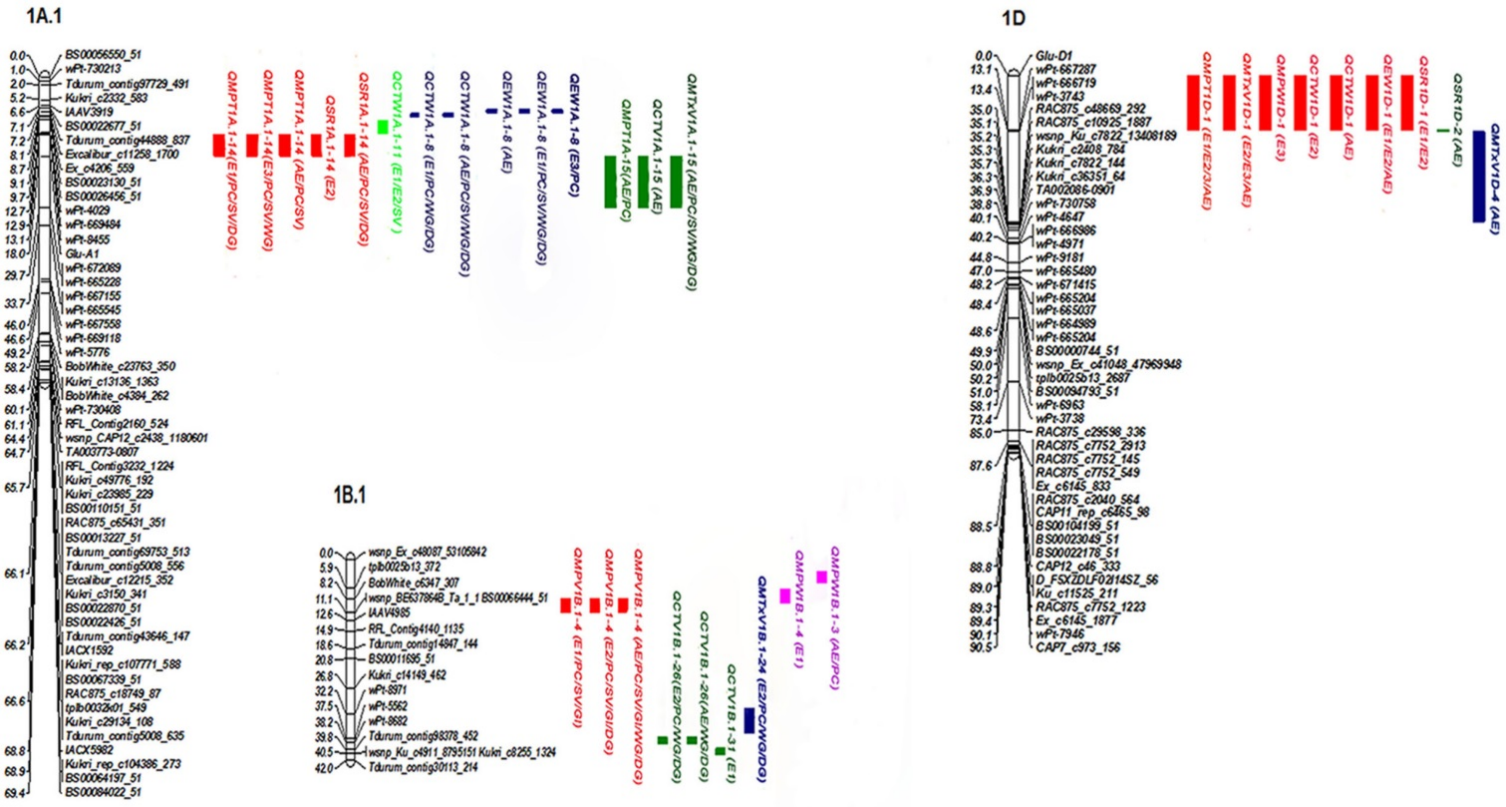
Most significant additive QTLs for mixing properties mapped in the RIL population based on unconditional and conditional QTLs.

Four QTLs for MPV were detected (primarily on 1B) with net additive effect values of 0.83-2.17%, and the phenotypic variation explained (PVE) value of 6.9-29.6% (Table 2, Figure 1). The effect value of *QMPV1B.1-4* was obviously changed when conditioned on SV, GI and PC, indicating that *QMPV1B.1-4* was partially influenced by SV and GI and PC; however, it was not detected when conditioned on WG and DG, indicating that it was completely influenced by them. The PVE of *QMPV1B.1-6* (PVE of 26.1%) was changed when conditioned on SV (PVE of 9%), but it showed no significant differences when conditioned on PC (PVE of 26.2%), GI (PVE of 25.8%) or DG (PVE of 25.6%), indicating that *QMPV1B.1-6* was not influenced by PC, GI or DG but was partially influenced by SV. Notably, *QMPV1B.1-24* was identified when conditioned on WG and DG in three environments, with PVEs of 8.9-27.1%. The positive alleles of the abovementioned QTL were derived from GC9411.

Six QTLs on chromosomes 1A, 1B, 3A and 5A were identified to associate with CTV, with PVEs of 1.1-21.4%. *QCTV1A.1-15* was not identified when conditioned on SV, GI, WG or DG, indicating that *QCTV1A.1-15* was completely influenced by the above related traits. *QCTV1B.1-31* showed positive additive effects with a higher PVE of 17.3%, indicating that favorable alleles were contributed by GC9411. *QCTV1B.1-26* was only identified when conditioned on PC, WG and DG, indicating that this QTL was detected when the influence of the mentioned traits was excluded. *QCTV5A.1-5* was only identified when conditioned on GI, with a PVE of 5.2%, indicating that *QCTV5A.1-5* was identified when the influence of GI was excluded. The favorable alleles of all the detected QTLs for CTV were contributed by GC9411.

Five QTLs associated with MTxV were identified on 1A, 1B and 1D; these QTLs were not related to DG, WG, or PC*.* Of these, *QMTxV1B-31* exhibited the maximum PVE of 16.4%, which was strongly influenced by GI and SV. *QMTxV1D-1* (*Glu-D1-WPT-667287*) was not identified when conditioned on SV, GI, DG or WG, with a PVE of 7.6-10.4%; this indicates that the QTLs associated with MTxV were completely influenced by SV, GI, DG and WG. *QMTxV1A.1-15* (*Glu-A1-WPT-672089*) was identified with a PVE of 8.07%; this QTL was not related to PC, SV, WG or DG, but it was crucially affected by GI.

Genotype-by-environment (G×E) interactions were investigated in the present study. Of the QTLs identified in the G×E analysis, *QMPV1B.1-4*, *QCTV1B.1-26* and *QMTxV1B.1-26* showed A×E% effects, with PVEs of 0.1%, 0.4% and 0.1%, respectively. Although the PVEs of the G×E QTLs were relatively low, these QTLs warrant attention for their possible role in wheat quality improvement 19.

#### Additive QTLs and genotype-by-environment interactions (G×E) for mixing properties MPW, CTW and EW

Important QTL clusters associated with MPW were identified on chromosomes 1B, 4B, and 1D (Table 3, Figure 1). *QMPW1B.1-4* was identified in unconditional analysis, with PVEs of 4.7-12.1% and net synergistic effects of 0.94-1.48%. The data indicated that cluster *QMPW1B.1-4* was completely influenced by SV, WG, and DG and was not detected when conditioned on these traits. *QMPW1B.1-24* was identified only in the conditional analysis, with PVEs of 6.1-15.9%, indicating that this QTL was identified when the influence of PC and WG was excluded. *QMPW4B.5-112* was identified only when conditioned on WG and DG. *QMPW1D-1*, with the PVE of 11.2%, was identified only when conditioned on WG and DG.

Five QTLs were identified as associated with CTW. These QTLs, with PVEs of 3.8-11.2%, were identified only in the conditional analysis, indicating that they were influenced by the quality and quantity of protein. *QCTW3B.3-31* was a stable QTL identified in multiple environments. *QCTW1D-1* (PVE of 13.04%) was detected in two environments, with an increased PVE compared with the corresponding unconditional QTL (unconditional PVE of 7.5%). *QCTW1D-1* was not identified when conditioned on SV, GI, DG or WG, indicating that *QCTW1D-1* was completely influenced by the above traits and was partially dependent on PC.

QTLs associated with EW were located on chromosomes 1A, 1D and 3B. *QEW1D-1* showed the greatest contribution to EW variation, with PVEs of 16.7%. The PVEs of* QEW1D-1* when conditioned on protein content did not differ significantly, and its PVE was decreased when conditioned on DG (PVE of 11.9%) compared to the corresponding unconditional QTL (unconditional PVE of 15.6%). *QEW1D-1* was not identified when conditioned on SV, GI or WG, demonstrating that this QTL was independent of PC, completely dependent on SV, GI and WG and partially influenced by DG. *QEW1A.1-8* was identified in multiple environments and showed PVEs of 6.63-10.04% in both conditional and unconditional mapping*. QEW3B.3-31* was the major QTL identified in multiple environments (E1 and AE), with PVEs of 2.4-11.2%; this QTL was independent of SV.

A G×E interaction of *QEW3B.3-31* was identified with an AE of 1.2%.

#### Additive QTLs and genotype-by-environment interactions (G×E) for mixing properties MPT, SR and IT

Four QTLs were detected as associated with MPT (Table 4, Figure 1). The PVE of *QMPT1A.1-14* was changed when conditioned on PC and SV, indicating that this QTL was partially influenced by PC and SV and completely influenced by GI.* QMPT1A.1-15* was not identified when conditioned on SV, GI, WG or DG.* QMPT1D-1* explained a PVE of 13.2-41.0%. Compared to the corresponding unconditional QTL, the PVE of* QMPT1D-1* was not significantly different when conditioned on PC, but it showed a high PVE of more than 20% when conditioned on SV, WG and DG. *QMPT1D-1* was not identified when conditioned on GI. The above results indicate that *QMPT1D-1* was completely influenced by GI, partially influenced by SV and gluten content, and not influenced by PC. *QMPT1A.1-14* and *QMPT1D-1* showed positive additive effects, indicating that favorable alleles were contributed by GC9411.

Four QTLs on chromosomes 1A, 1B, 1D and 2B were discovered to be related to SR. Among them, *QSR1A.1-14* was partially influenced by PC, SV and DG, whereas it was completely dependent on GI and WG. *QSR1B.1-6* and *QSR2B.7-34* were identified in multiple environments with a high PVE of more than 10%, indicating that these QTLs were major loci with strong stability. *QSR1D-1*, which was identified by both conditional and unconditional QTL mapping, was influenced by PC. The favorable alleles of all the QTLs, except for *QSR1B.1-6* and *QSR2B.7-34*, were contributed by GC9411.

One major QTL cluster on chromosome 1B was identified as associated with IT. The three QTLs were located close to each other, with a maximum PVE of 40.9% and a net additive effect of 15.9% min. *QIT1B.1-4* was detected only when conditioned on GI, WG and DG, which should attract more attention in breeding programs.

The abovementioned QTLs for mixing properties were mainly distributed on chromosomes 1A, 3A, 5A, 1B, 2B, 7B and 1D. Among them, the favorable alleles were mostly contributed by GC9411.

## Discussion

Although several QTLs for mixing properties have been identified in previous research, there is no consensus on the merits of mixing properties in the selection of optimal dough properties. Furthermore, most of these studies only focused on additive effects using unconditional QTL mapping methods without considering the epistatic or G×E interaction effects. In this study, additive and epistatic-interaction QTLs for mixing properties, as well as their environmental interaction effects, were analyzed. In addition, we combined conditional and unconditional QTL analyses to investigate the molecular mechanisms governing mixing properties.

### Important QTLs associated with mixing properties

According to the present results, several QTL clusters were identified with high PVEs and strong stability, particularly those distributed on chromosomes 1A, 1B and 1D (Fig. 1). *Locus 1A.1-14* within the genomic region *WPT-8455-GLU-A1* was detected in multiple environments; this QTL was related to MPT and SR and explained a PVE of 7.41-12.69%. The effect was speculated to be closely related to *Glu-A1*. A comparison of map positions revealed that *1A.1-8* was 13.9 cM away from *1A.1-14* (*WPT-8455-GLU-A1*), which was predicted to be different from *Glu-A1*. Further investigations are needed to completely rule out any such possibility.

QTLs related to mixing parameters (MPV, CTV, MTxV, MPW, SR, and IT) were detected on chromosome 1B and have been reported in several previous studies 2-4. Here, using SDS-PAGE analysis, we confirmed that GC9411 and SN01-35 have the same HMW glutenin subunits, 7+8, which are controlled by *Glu-B1* on 1BL. Therefore, the QTLs on chromosome 1BS identified as controlling mixing properties in the present study are most likely not associated with the *Glu-B1* locus in the population. Consensus results were obtained by Echeverry-Solarte 20 and Liu 16, who detected loci for increased IT located on 1BS. Further investigations are needed.

Furthermore, two major pleiotropic QTL clusters independent of *Glu-B1* were detected on 1BS. One cluster, including *1B.1-24* and *1B.1-26*, was associated with MPV and CTV. The other cluster, containing *1B.1-3* and *1B.1-4*, was identified as associated with MPV and MPW and IT in multiple environments*.* In addition,* 1B.1-6* and* 1B.1-26* were detected in a single environment, of which *1B.1-6* and *1B.1-26* explained higher phenotypic variation. One major cluster that included three major QTLs (*QIT1B.1-3*, *QIT1B.1-4* and* QIT1B.1-6*) for IT was located on chromosome 1B. Its maximum PVE was 40.9%, with a net additive effect of 40.9%, and its favorable alleles were contributed by GC9411. This cluster warrants greater attention in breeding programs.

The genomic region most consistently associated with MPT, MTxV, MPW, CTW, EW and SR were identified on chromosome 1D, especially *1D.1-1* (*GLU-D1-WPT-667287*), which was detected in all environments, with PVEs of 14.9%-20.7%. The high PVEs of these QTLs and their stability across environments further confirm the importance of HMW-GS encoded by* Glu-D1*. The closest region to these QTLs was previously identified as associated with mixing properties and other quality traits 3, 4, 21, 22, 23.

The high-molecular-weight glutenin subunit (HMW-GS) composition of Shannong01-35 was null, 7+8, 2+12; that of Gaocheng9411 was 1, 7+8, 5+10. The effect of Glu-D1 on quality was mainly due to the difference between HMW-GS 2+12 and 5+10. Lefebvre et al. (2000) showed that large gluten compositions dominated dough rheology 24, and glutenin subunit 5+10 was larger than 2+12 25. Many researchers have pointed out that subunit 5+10 had better quality characteristics of strong dough strength and food quality 26, 27. Not surprisingly, GC9411 exhibited higher mixing parameter values than SN01-35 in all environments.

### Conditional QTLs for mixing properties

Conditional analysis has the ability to discern the contribution of each component trait to a complex trait. Based on the QTL analysis of mixing properties, locus* 1A.1-14*, which controlled MPT and SR, was identified as partially influenced by PC, SV, and DG and completely influenced by GI. An important QTL, *1D-1*, that was associated with MPT, EW, CTW and MTxV was completely influenced by GI, partially influenced by SV, WG and DG, and slightly influenced by PC. A QTL that controlled MPV and MPW,* 1B.1-4*, was partially influenced by PC, SV, and GI and was crucially affected by WG and DG. It is worth noting that *1B.1-24*, an additional locus controlling MPV and MPW, was identified in multiple environments when the influence of the examined traits was excluded, but it was not identified in the unconditional analysis. *QCTV1A.1-15*, which was completely influenced by protein-related traits, was detected only in unconditional mapping. *QCTV5A.1-5* was identified only when the influence of GI was excluded.

*QMPW1B.1-4* was partially influenced by SV, PC, GI, and WG. QTL clusters *4B.5-59*, *4B.5-58*, and *4B.5-60* were partially influenced by all the examined traits. *QCTW3B.3-31* and *QCTW6B.5-409* were identified only in the conditional analysis. *QEW1D-1* was completely influenced by SV and GI. Only two QTLs (*QSR1B.1-6* and* QSR2B.7-34*) were found to be partially influenced by protein-related traits.

## Conclusion

*1A.1-15* and *1D.1-1* were major loci identified as associated with rheological properties influenced only by SV and GI. This finding demonstrates that these two QTL loci provide an important contribution to wheat quality at the genetic level. The combination of conditional and unconditional QTL mapping in the present study provided an opportunity for the detection of the mechanisms underlying the mixing properties of wheat.

## Figures and Tables

**Table 1:** Phenotypic values for mixographic parameters of the RIL population in different environments.

Trait	Env	SN01-35	GC9411	Min	Max	Mean	CV%	*H*^2^ (%)
MPT	E1	1.8	3.5	1.5	4.3	2.5	22.62	57.82
	E2	1.5	5.5	1.5	6.3	3.2	32.16	
	E3	1.6	4.9	1.7	5.6	2.9	25.26	
	AE	1.6	4.6	1.5	4.9	2.9	24.94	
MPV	E1	63.1	67.8	50.9	76.5	67.0	6.87	65.23
	E2	54.9	51.7	41.2	59.7	52.6	6.47	
	E3	56.4	57.4	48.0	64.9	55.9	6.11	
	AE	58.2	59.0	47.9	70.5	58.8	5.98	
CTV	E1	20.7	28.3	20.4	61.9	51.7	9.95	49.89
	E2	35.2	48.3	30.9	48.7	43.0	7.12	
	E3	40.5	47.8	30.8	50.5	43.1	7.61	
	AE	32.1	41.5	33.0	56.1	46.2	7.27	
MTxV	E1	48.4	57.7	39.2	63.8	53.6	8.12	67.13
	E2	36.3	50.2	32.9	50.1	44.5	6.82	
	E3	41.7	50.2	35.0	53.5	44.7	7.12	
	AE	42.1	52.7	36.0	58.1	47.8	6.87	
MPW	E1	20.7	28.3	5.2	40.6	25.3	19.89	62.19
	E2	15.1	18.4	9.5	23.5	17.3	15.59	
	E3	21.1	18.1	12.2	30.9	19.4	18.48	
	AE	19.0	21.6	9.9	28.9	20.9	13.70	
CTW	E1	3.9	11.1	3.2	51.8	6.7	80.32	53.18
	E2	2.9	11.9	2.5	13.9	6.0	36.01	
	E3	3.1	6.6	2.9	17.6	5.0	35.64	
	AE	3.3	9.9	3.3	20.3	5.9	41.86	
EW	E1	4.2	14.2	3.3	21.6	7.2	49.27	49.82
	E2	3.1	13.7	3.0	14.0	6.8	37.54	
	E3	3.4	10.0	3.1	13.1	5.3	37.83	
	AE	3.6	12.6	3.3	16.8	6.5	38.87	
SL	E1	-	8.3	-0.6	15.7	7.3	58.24	50.97
	E2	-	1.2	0.2	9.9	4.5	46.13	
	E3	-	4.6	1.1	16.3	6.1	39.46	
	AE	-	4.7	1.1	16.3	6.1	41.76	
SR	E1	-3.1	-3.5	-5.6	-0.5	-3.1	30.20	53.85
	E2	-1.7	-1.1	-3.6	-0.4	-2.0	36.97	
	E3	-2.1	-2.1	-4.1	-0.4	-2.5	26.82	
	AE	-2.3	-2.3	-3.8	-0.9	-2.5	23.30	
IT	E1	515.2	585.9	430.8	661.7	565.3	7.25	62.10
	E2	405.3	470.5	356.4	506.3	458.7	5.69	
	E3	448.7	489.6	403.5	544.9	472.3	5.25	
	AE	456.4	515.3	398.7	605.4	500.9	5.75	

In the environment column, E1, E2, E3 refer to 2009 Tai'an, 2010 Tai'an, and 2010 Suzhou; AE refer to the average data of three environments. MPT, midline peak time; MPV, midline peak value; CTV, curve tail value; MTxV, midline time-8 min value; MPW, midline peak width; EW, end width; CTW, curve tail width; SL, left slope of peak; IT, integral of curve tail; CV, coefficients of variation; *H^2^*, the broad-sense heritability.

**Table 2:** Unconditional and conditional additive QTLs for mixing parameters MPV, CTV, MTxV.

Trait	QTL	E	Flanking markers	Position	A^a^	Unconditional PVE (A)%b	PVE (AE)%c	Conditional PVE (A)%^d^				
								PC	SV	GI	WG	DG
MPV	*QMPV1B.1-4*	E1/E2/AE	BOBWHITE_C6347_307-WSNP_BE637864B_TA_1_1	9.2	-0.83	22.3/19.5/-	0.1	29.6/30/28.9	15.9/12.6/14.6	6.9/15.9/21.1	-/-/7.5	-/25.5/21.4
	*QMPV1B.1-6*	E3	BS00066444_51-IAAV4985	11.1	-1.58	26.1	-	26.2	9.0	25.8	22.7	25.6
	*QMPV 1B.1-24*	E1/E2/AE	WPT-8971-WPT-5562	36.2/-	-2.17	-	-	-	-	-	26.5/17.5/8.9	27.1/-/-
	*QMPV 7B.4-22*	E1	RAC875_REP_C72877_159-WPT-5463	20.1	-1.23	13.0	-	11.2	-	-	-	-
CTV	*QCTV1A.1-6*	AE	BS00022677_51-TDURUM_CONTIG44888_837	7.1	-0.61	4.57	-	-	-	-	-	-
	*QCTV1A.1-15*	AE	GLU-A1-WPT-672089	22	-1.17	7.00	-	-	-	-	-	-
	*QCTV 1B.1-26*	E2/AE	WPT-8682-TDURUM_CONTIG98378_452	38.2	-	-	0.4	20.6			21.4	20.9
	*QCTV 1B.1-31*	E2	KUKRI_C8255_1324-TDURUM_CONTIG30113_214	41.5	-1.36	17.3	-	-	-	-	-	-
	*QCTV 3A.2-236*	E3	EXCALIBUR_C31571_136-XGPW8072	123.3	-0.67	1.1	-	-	-	-	-	-
	*QCTV 5A.1-5*	AE	BS00001525_51-BOBWHITE_C31599_604	14.4	-0.6	-	-	-	-	5.2	-	-
MTxV	*QMTxV 1A.1-15*	AE/E1	GLU-A1-WPT-672089	22	-1.36/-	-8.07/-	-	7.34/5.73	8.29/6.18	-	6.25/5.52	6.59/5.65
	*QMTxV1B.1-26*	E2	WPT-8682-TDURUM_CONTIG98378_452	39.2	-1.98	-	0.1	20.5	-	-	21.4	21
	*QMTxV 1B.1-31*	E3	KUKRI_C8255_1324-TDURUM_CONTIG30113_214	41.5	-1.29	16.4	-	13.7	-	-	14.6	14.4
	*QMTxV 1D-1*	E2/E3/AE	GLUD1-WPT-667287	11	-1.04/-1.112/-1.02	10.4/11.5/ 12.0	-	7.6/-/-	-	-	-	-
	*QMTxV 1D-4*	AE	WPT-3743-RAC875_c48669_292	15.4	-0.98	-	-	-	-	-	7.2	0.92

^a^ Additive effect of the QTL. A positive number indicates that the Shannong01-35 allele was associated with larger trait values than the Gaocheng9411 allele; a negative number indicates that the Gaocheng9411 allele was associated with larger trait values than Shannong01-35; ^b^ Phenotypic variance explained by additive effects in unconditional analysis; ^c^ Phenotypic variance explained by additive-by-environment interaction effects; ^d^ Phenotypic variance explained by additive effects in conditional analysis. PC, Protein content; SV, sedimentation volume; GI, gluten index; WG, wet gluten content; DG, dry gluten content.

**Table 3:** Unconditional and conditional additive QTLs for mixing parameters MPW, CTW, and EW.

Trait	QTL	E	Flanking markers	Position	A^a^	Unconditional PVE (A)%^b^	PVE (AE)%^c^	Conditional PVE (A)%^d^				
								PC	SV	GI	WG	DG
MPW	*QMPW1B.1-4*	E1/E3/AE	BOBWHITE_C6347_307-WSNP_BE637864B_TA_1_1	8.2	-0.94/-1.48/-1.0	12.1/-/4.7	-	-/19.9/8.3/-	-	-	-	-
	*QMPW1B.1-5*	E3	WSNP_BE637864B_TA_1_1-BS00066444_51	11.1	-1.66	12.1	-	12.1-	-19.7	-20.2	-	20.1-
	*QMPW 1B.1-24*	E2/AE	WPT-8971-WPT-5562	32.2	-1.01/-	-	-	14.7/-	-/14.6/-	-/14.3	15.0/12.5	-/15.9
	*QMPW1D.1-1*	E3	GLU-D1-WPT-667287	9.0	1.25	-	-	11.2	-	-	-	-
	*QMPW 4B.5-59*	E2	RAC875_C2545_1127-WSNP_EX_C12526_19951640	13.9	1.0	13.80	-	-	-	-	-	-
	*QMPW 4B.5-58*	E2	RA_C41921_1056-RAC875_C2545_1127	13.9	1.18	-	-	-	20.3	-	-	-
	*QMPW 4B.5-60*	E2	WSNP_EX_C12526_19951640-TDURUM_CONTIG67477_136	13.9	1.01	-	-	-	-	13.4	-	-
	*QMPW4B.5-112*	E2	TDURUM_CONTIG42107_2206-WSNP_EX_C16825_25387841	65.8	0.78	-	-	-	-	-	9.5	10.9
CTW	*QCTW1A.1-8*	E1/AE	EXCALIBUR_C11258_1700-EX_C4206_559	8.1	-0.83	-	-	8.64	-	-	6.93	7.27
	*QCTW1 A.1-11*	E1/E2	BS00026456_51-WPT_4029	9.7	-0.77	-	-	9.36/6.68	-	-	-	-
	*QCTW1D-1*	E2/AE	GLU-D1-WPT-667287	11.0/6	-0.92/-0.86	-7.5/2.5	-	-/12.35	-	-	-	-
	*QCTW 3B.3-31*	E1/AE/E2	BS00064177_51-EXCALIBUR_C25566_423	33.3	-0.93/-0.57/-0.57	-	-	11.1/7.3/5.8	-	-	9.8	10.5/ 3.8/11.2
	*QCTW6B.5-409*	AE	TDURUM_CONTIG30640_263-WSNP_CAP11_C1541_857160	99	0.52/0.52	-	-	4.6/-	-/4.6	-	-/3.8	-/4.0
EW	*QEW1A.1-8*	E1/E2/AE/E3	EXCALIBUR_C11258_1700-EX_C4206_559	8.1	-0.70/-	7.56/-	-	9.05/8.07/6.31/5.99	-/10.04/ 8.29/6.63	-/1.81	-/7.49/ 6.31/4.43	-/7.86/ 6.63/5.13
	*QEW1D-1*	E1/E2/ AE	GLU-D1-WPT-667287	7	-1.60/-0.96/-1.16/-0.93	15.6/11.4/ 16.5/10.6	-	16.5/13.0/16.7/-	-	-	-	11.93/ 11.2/-
	*QEW3B.3-31*	E1/AE	BS00064177_51-EXCALIBUR_C25566_423	33.3	-/-0.83	-/10.70	-/1.2	11.2/ 2.4	-	-	9.6/-	10.3/-

^a b c d^ Same as Table 2.

**Table 4:** Unconditional and conditional additive QTLs for mixograph parameters MPT, SR and IT.

Trait	*QTL*	E	Flanking markers	Position	A^a^	Unconditional PVE (A)%^b^	PVE (AE)%^c^	Conditional PVE (A)%^d^				
								PC	SV	GI	WG	DG
MPT	*QMPT1A.1-14*	*AE/E1/E3*	WPT-8455-GLU-A1	16.1	-0.35/-/-	11.14/-	-	12.69/*14.36*/*13.84*	12.05/14.13/ *15.76*	-	-/-/9.04	-/7.23/-
	*QMPT1A.1-15*	*AE*	GLU-A1-WPT-672089	37.2	-0.17	-	-	5.2	-	-	-	-
	*QMPT1A.1-24*	*E2*	KUKRI_C13136_1363-BOBWHITE_C4384_262	58.4	-0.27	6.93	-	-	6.73	-	-	-
	*QMPT1D-1*	E1/E2/E3/AE	GLU-D1-WPT-667287	11	-0.35/-0.67 /-0.45/-0.47	33.4/27.1/33.7/ 36.7	-	37.9/33.0/ 35.5/41.0	20.7/-/27.1/ 26.7	-/-/-/13.2	14.9/-/22.2/ 28.9	20.4/-/ -/31.25
SR	*QSR1A.1-14*	E2/AE	WPT-8455-GLU-A1	16.1	-0.29/-0.25/-	7.41/8.10		-/9.47	8.50/3.90	-	-	5.82/-
	*QSR1B.1-6*	E3	BS00066444_51-IAAV4985	11.1	0.2	8.4	-		7.8/-	10.8/3.2	-	-
	*QSR1D-1*	E1/E2	GLU-D1-WPT-667287	10	-0.31/-0.26	-/11.5		9.5/15.5	-/11.7	-	-	-
	*QSR 2B.7-34*	E1/AE	EXCALIBUR_C181_265-EXCALIBUR_REP_C107306_130	32.7	0.31/0.21	-	-	-	11.3/12.1/-	-	-	-/3.2
IT	*QIT 1B.1-3*	AE	TPLB0025B13_372-BOBWHITE_C6347_307	6.9	-15.92	30.6	-	40.9	-	-	-	-
	*QIT 1B.1-4*	E1/E3/AE	BOBWHITE_C6347_307-WSNP_BE637864B_TA_1_1	10.2	-13.36/-	-	-	-	15.2/-/16.7	25.2/-/23.0	-	-/12.9/17.7
	*QIT 1B.1-6*	E3	BS00066444_51-IAAV4985	11.1	-11.6	21.9	-	13.8	-	11.5	18.8	12.9

^a b c d^ Same as Table 2.
